# Diffusion-Weighted MRI in the Assessment of Early Treatment Response in Patients with Squamous-Cell Carcinoma of the Head and Neck: Comparison with Morphological and PET/CT Findings

**DOI:** 10.1371/journal.pone.0140009

**Published:** 2015-11-12

**Authors:** Eduardo Bruno Lobato Martins, Rubens Chojniak, Luis Paulo Kowalski, Ulisses Ribaldo Nicolau, Eduardo Nóbrega Pereira Lima, Almir Galvão Vieira Bitencourt

**Affiliations:** 1 Department of Imaging, A.C.Camargo Cancer Center, São Paulo, SP, Brazil; 2 Department of Head and Neck, A.C.Camargo Cancer Center, São Paulo, SP, Brazil; 3 Department of Oncology, A.C.Camargo Cancer Center, São Paulo, SP, Brazil; National Cancer Centre Singapore, SINGAPORE

## Abstract

**Objective:**

To evaluate changes in apparent diffusion coefficients (ADC) as measured by magnetic resonance imaging (MRI) before and after the treatment of primary tumors and cervical metastases in patients with squamous-cell carcinoma (SCC) of the head and neck, and to compare these values to the results of widely used morphological criteria and [18F]-FDG PET/CT findings.

**Material and Method:**

This was a longitudinal, prospective, single-center nonrandomized trial involving patients with head and neck SCC treated with chemotherapy alone or in combination with radiotherapy. Imaging examinations ([18F]-FDG PET/CT and diffusion-weighted MRI) were performed on the same day, up to one day prior to the beginning of the first treatment cycle, and on the 14th day of the first chemotherapy cycle. Treatment response was evaluated based on the Response Evaluation Criteria in Solid Tumors (RECIST) and World Health Organization (WHO) morphological criteria, as well as PET Response Criteria in Solid Tumors (PERCIST) metabolic criteria.

**Results:**

Seventy-five lesions were examined in 23 patients. Pre- and post-treatment comparisons of data pertaining to all target lesions revealed reductions in tumor size and SUV, as well as increases in ADC values, all of which were statistically significant. The increase in ADC following treatment was significantly higher in patients classified as complete responders by both morphological criteria than that observed in any of the other patient groups of response. Patients with a complete metabolic response also showed greater increases in ADC values as compared to the remaining groups.

**Conclusion:**

The assessment of tumor response based on diffusion-weighted MRI showed an increase in the ADC of cervical lesions following treatment, which was corroborated by morphological and metabolic findings. Associations between changes in ADC values and treatment response categories using morphologic criteria and [18F]-FDG PET/CT were only identified in complete responders.

## Introduction

Squamous-cell carcinoma (SCC) is the most common type of head and neck cancer, accounting for 95% of tumors in these locations. The treatment of head and neck cancers is highly complex and varies according to tumor location, accessibility, and extension to adjacent organs and tissues.^1^ Initial tumors are usually treated by surgery or radiotherapy, while advanced tumors are treated with different combinations of surgery, radiotherapy and chemotherapy.[1]

In recent years, chemotherapy has played an increasingly important role in the treatment of locally advanced cancers, and has proven effective in several different applications, such as induction or neoadjuvant, concomitant or adjuvant to radiotherapy, as well as isolated or in combination with other treatments. A meta-analysis by Pignon et al. published in the year 2000 found the combination of chemotherapy and locoregional treatment to be effective in the management of locally advanced head and neck tumors.[2] Clinical treatment with chemotherapy and radiotherapy are often combined as an initial treatment strategy for organ preservation, with surgical resection being reserved for non-responders to this therapy.

The effectiveness of clinical treatments is traditionally evaluated using response criteria based on morphological findings such as imaging-based tumor size measurements. The most common such criteria are the Response Evaluation Criteria in Solid Tumors (RECIST) and World Health Organization (WHO) response guidelines, and their variants.[3] However, in recent years, new methods have also been developed for the functional assessment of tumors, the most promising of which appear to be fluorine-18 fluorodeoxyglucose positron emission tomography—computed tomography ([18F]-FDG PET/CT) and diffusion-weighted magnetic resonance imaging (MRI). Although [18F]-FDG PET/CT is already widely used in the clinical practice of head and neck oncology,[4] the use of diffusion-weighted imaging is still limited to research settings.

Diffusion-weighted MRI is based on the random movement of water molecules in human tissue. The movement of water molecules is restricted in hypercellular environments such as some types of tumor tissue.[5–6] The mobility of water molecules is quantified by the apparent diffusion coefficient (ADC). In oncology, this method has been used to diagnose and differentiate between benign and malignant lesions, to detect cancer recurrence and, more recently, to assess treatment response in different organs or tissues.[7–8]

Although some papers have showed that there is an increase in ADC after treatment, only few have evaluated early response to non-surgical treatment comparing to widely used tools. The aim of this study was to assess early changes in ADC measurements obtained by MRI before and after the treatment of primary tumors and cervical metastasis in patients with head and neck SCC treated with chemotherapy alone or associated with radiotherapy. The changes in ADC values were compared to alterations in patient response classification according to morphological criteria and metabolic activity as detected by [18F]-FDG PET/CT.

## Materials and Methods

This was a longitudinal, prospective, single-center nonrandomized study involving patients with stage III and IV (no evidence of distant metastasis) head and neck SCS, referred to neoadjuvant chemotherapy followed by radiotherapy, chemoradiotherapy, or adjuvant chemotherapy and concomitant radiotherapy. This study was approved by the Antônio Prudente Foundation / AC Camargo Cancer Center’s Institutional Research Board (n. 1486/10) and all patients provided written consent before enrollment. Pregnant or lactating women, as well as patients with contraindiciations for MRI (e.g. pacemakers, metallic implants or claustrophobia) or other primary tumors (except for adequately treated basal cell skin cancer) were excluded from the sample.

Imaging examinations for tumor staging ([18F]-FDG PET/CT and diffusion-weighted MRI) were performed up to one day prior to the first chemotherapy cycle and on the 14th day of the first cycle to assess early treatment response. Diffusion MRI was performed on the same day as [18F]-FDG PET/CT scans. Drugs used in neoadjuvant chemotherapy for head and neck cancer exhibit high toxicity, therefore the early identification of non-responders, after 14 days of the first cycle, could prevent these patients from being subjected to two more cycles of these drugs, sparing its toxicity, and send them directly to adjuvant radiation and chemotherapy.

Seventy-five separate tumors were examined in 23 patients recruited between January 2010 and October 2012, including 23 primary tumors and 52 metastatic lymph nodes. The sample was predominantly male (n = 21; 91%) and had a mean age of 56 (46–69) years. Four of the 23 patients only underwent one staging examination due to death or loss to follow-up. Therefore, the analysis of treatment response according to morphological criteria, which required complete patient data, was performed on a sample of 19 patients. Imaging exams were also used to evaluate the size, ADC and SUV of each tumor (n = 75), compare these values to those of normal tissue, and calculate changes in these parameters between pre- and post-treatment assessments.

### Diffusion-weighted MRI

MRI was performed on a 1.5-T scanner (Signa Excite HDX; GE Healthcare, Milwaukee, USA) using a cervical coil. Before the diffusion-weighted sequences, conventional axial T2-weighted spin-echo images with fat suppression (TR/TE 3800/100 ms; 288 x 192 matrix; FOV: 240 x 210 mm; NEX: 2; 4 mm slice thickness; 1 mm interval), Coronal T2 with fat supression (same pulse parametres and matrix 320 x 224 and FOV: 240 x 200 mm) and axial T1-weighted images (TR/TE 490/9,6 ms; 320 x 224 matrix; FOV: 240 x 210 mm; NEX: 4; 4 mm slice thickness; 1 mm interval) were also obtained. Diffusion-weighted single-shot echo-planar imaging (EPI) sequences in the axial plane were obtained using the following parameters: b = 0 and 1000 s/mm2; TR/TE 2000/75 ms; 256 x 256 matrix; FOV: 230 mm; NEX: 16; 4 mm slice thickness; 0 mm interval.

Images were transferred to the workstation (Advantage Windows version 4.2_07; GE Healthcare, Milwaukee, USA) and diffusion images were post-processed using the Functool software (GE Healthcare, Mialwaukee, USA). Diffusion sequences were used to provide ADC maps (black/white and in color) for each tumor using both b values.

Images were submitted to region-of-interest (ROI) analysis on a slice per slice basis for the evaluation of diffusion statistics. ADC values were calculated based on actual tumor size and viable tumor regions within the ROI as interpreted by a radiologist specialized in head and neck MRI. Liquefied areas suggestive of tumor necrosis were discarded from the analysis. Minimum and mean ADC values were calculated and compared between the primary tumor, lymph nodes and normal tissue (healthy pharyngeal mucosa). Mean ADC was used for analysis because of the high variance observed on minimum ADC.

The ADC of each tumor was automatically measured by the post-processing software after the determination of ROI using the following equation: ADC = −(In (Sh/Si)) / (bh−bi). Sh and Si represent the signal intensity in the ROI calculated based on the difference between the two b-values (bh and bi). In the present study, the maximum b-value (bh) was 1000 sec/mm^2^, and the minimum (bi), 0 sec/mm^2^.

### [18F]-FDG PET/CT

Image data were obtained using a Gemini PET/CT scanner (Philips Medical Systems). Patients fasted for 6 hours before the examination. PET-CT was performed after administration of 0.154 mCi / kg of [18F]-FDG, which was injected intravenously through a peripheral venous access. Before administration of 18F-FDG serum glucose levels were below 150 mg/dl. [18F]-FDG PET/CT images were obtained approximately 90 minutes after the injection of [18F]-FDG (range between 60 and 120 minutes). The complete examination, from the time of image acquisition to the evaluation of image quality, lasted approximately 25 minutes. All patients underwent non-contrast computed tomography (CT) followed by a positron emission tomography (PET) scan from the head to midthigh level. [18F]-FDG PET/CT images were analyzed for areas of abnormal ^18^FDG uptake, whose radiotracer levels were compared to standard [18F]-FDG uptake or maximum SUV in the anomalous areas found. The SUV was calculated using the concentration of 18F-FDG in the tissue and corrected for injected dose, injection time and the body surface of the patient.

Reduced metabolic activity was evaluated based on decreases in SUV from pre- to post-treatment and on the SUV measurements obtained in each [18F]-FDG PET/CT examination. Changes in SUV determined by the [18F]-FDG PET/CT examinations were used to classify patients according to PERCIST criteria. A complete metabolic response (CMR) was defined as the complete normalization of SUV in all tumors, with mean lesion SUV being similar to that of the surrounding tissue. Progressive metabolic disease (PMD) was defined as an increase of at least 30% in the mean SUV of target lesions. A reduction of at least 30% in the mean SUV of target lesions was considered a partial metabolic response (PMR), while an increase or decrease in SUV which did not exceed 30% was indicative of stable metabolic disease (SMD).[9]

### Morphological Response Criteria

Patients were classified and compared based on RECIST and WHO criteria. Tumor size was measured in the T2-weighted sequence of the same MRI exams performed for diffusion-weighted evaluation up to one day prior to the first chemotherapy cycle and on the 14th day of the first cycle.

WHO criteria classify patients in the following manner, according to the bidimensional product of tumor dimensions:

- Complete response (CR)—Disappearance of all known lesions, determined clinically, radiologically or by endoscopy/biopsy.
- Partial response (PR): in case of bidimensionally measurable disease, reduction of at least 50% in the product of the largest perpendicular diameters of all measurable lesions. In the case of unidimensionally measurable lesions, PR is defined by a reduction of at least 30% in the sum of diameters of all measurable tumors. Although not all lesions must have decreased in size, no tumors must have grown or appeared during the evaluation period.
- Stable disease (SD): in case of bidimensionally measured disease, <50% decrease and <25% increase in the sum of products of the largest perpendicular diameters of all measurable lesions detected before treatment. In case of unidimensionally measurable disease, <50% decrease and <25% increase in the sum of diameters of all measurable tumors detected before treatment. No new lesions must be present.
- Progressive disease (PD): >25% increase in the size of at least one measurable lesion as compared to baseline.

RECIST 1.1 criteria were calculated based on the largest diameter of the primary tumor and the short axis of metastatic lymph nodes (two to three target lesions were included for each patient—maximum number per organ). A CR was considered the disappearance of all known lesions, while a PR was characterized by a decrease of 30% in the product of the maximum diameters of target lesions, PD was defined as an increase of 20% in the product of the diameters of target lesions combined with the appearance of new lesions, and the failure to meet criteria for PR or PD was considered SD.

### Statistical Analysis

Target variables were expressed as absolute and relative frequencies, or summarized using standard descriptive statistics (mean, median, standard deviation and minimum and maximum values). Student's T-tests (or non-parametric Mann-Whitney tests, when necessary) were used for between-group comparisons of scalar variables. Categorical variables were compared using Pearson's Chi-square test with Yates correction, or Fisher's Exact Test.

Given the occurrence of multiple tumors per patient, the changes in ADC values between pre- and post-treatment measurements were evaluated using repeated measures analysis of variance, as described by Pinheiro and Bates (2000).[10] ROC (Receiver operating curve) analysis was used to classify lesions as benign or tumoral. Data were analyzed using the R software, version 2.15.2 (www.r-project.org), and significance was set at 5%.

## Results

### Patient Characteristics

Most primary tumors were located in the oropharynx (60%), while 17% were in the larynx, 9% in the hypopharynx, 9% in multiple sites (oropharynx and oral cavity) and 4% in unknown primary sites. All patients had at least one enlarged cervical lymph node, bilateral involvement were observed in 48% of patients, while 52% had unilateral involvement. Most patients had stage IV cancer (74%), while 26% were classified into stage III (Table 1).

**Table 1:** Age, gender, primary tumor site, clinical stage and location of affected lymph nodes in patients included in the study.

Patient ID	Age	Gender	Primary tumor site	Stage		Side of affected lymph nodes
1	47	M	Oropharynx (L tonsil)	T4N1MO	IVa	Bilateral
2	54	M	Oropharynx (R tonsil)	T3N3M0	IVb	Bilateral
3	50	M	Oropharynx (bilateral base of the tongue)	T4N2bM0	IVa	Bilateral
4	48	F	Bilateral subglottic larynx	T3N2M0	IVa	Right
5	65	M	Oropharynx (R base of the tongue)	T3N2bM0	IVa	Bilateral
6	55	M	Oropharynx (L tonsil)	T2N2bM0	IVa	Bilateral
7	59	M	Oropharynx (R tonsil)	T3N1M0	III	Right
8	60	M	Oropharynx (bilateral base of the tongue)	T2N2cM0	IVa	Bilateral
9	57	M	R supraglottic larynx	T3N2cM0	IVa	Bilateral
10	55	M	Oropharynx (L base of the tongue)	T2N3MO	IVb	Left
11	58	M	Oropharynx (L tonsil)	T1N2cM0	IVa	Left
12	51	M	Oropharynx (R tonsil)	T3NIM0	III	Right
13	68	M	Oropharynx (L tonsil)	T3N1M0	III	Left
14	53	M	Oropharynx (R tonsil)	T3N2bM0	IVa	Right
15	46	M	Oropharynx (R base of the tongue)	T3N1M0	III	Right
16	59	M	L glottic larynx	T3N1M0	III	Right
17	53	F	L supraglottic larynx	T3N1M0	III	Bilateral
18	53	M	Oropharynx (R tonsil)	T4N2M0	IVa	Bilateral
19	60	M	Unknown	TXN3M0	IVb	Right
20	50	M	Oropharynx (bilateral base of the tongue)	T4N3M0	IVb	Bilateral
21	57	M	Hypopharynx (L pyriform sinus)	T3N2M0	IVa	Left
22	60	M	Oropharynx (bilateral base of the tongue)	T4N3M0	IVb	Bilateral
23	69	M	Hypopharynx (R pyriform sinus)	T3N2bM0	IVa	Right

All patients received chemotherapy, with 70% receiving neoadjuvant and adjuvant chemotherapy combined with radiotherapy, 30% receiving only adjuvant chemotherapy and concomitant radiotherapy, and two receiving neoadjuvant chemotherapy only, since death occurred before the start of the adjuvant treatment. Most patients (91%) were treated with radiotherapy, with 61% receiving intensity-modulated radiation therapy (IMRT) (), 33% receiving 3D radiotherapy, and 5%, 2D radiotherapy. Only 3 patients (14%) underwent surgical resection.

### Lesion Data

The mean greatest axial dimension of target lesions on baseline examination was 3.4 cm (0.8–8.0cm), while that of the smallest axial dimension was 2.3 cm (0.5–7.6cm). The mean greatest lymph node dimension was was 2.3 cm (0.8–6.3cm), while the mean short-axis diameter was 1.7cm (0.6–5 cm).

The analysis of SUV values revealed that the mean maximum SUV in primary lesions was 8.1 (2.6–15.1), with a standard deviation of 3.2. Mean SUV in the lymph nodes was 4.7 (1.3–17.1), with a standard deviation of 3.2.

The mean ADC in primary tumors was 0.91 (0.68–1.31) x 10^−3^ mm^2^/s, with a standard deviation of 0.14 x 10^−3^ mm^2^/s. The mean SUV in lymph nodes was 0.85 (0–1,26) x 10^−3^ mm^2^/s, with a standard deviation of 0.19 x 10^−3^ mm^2^/s.

**Fig 1:**
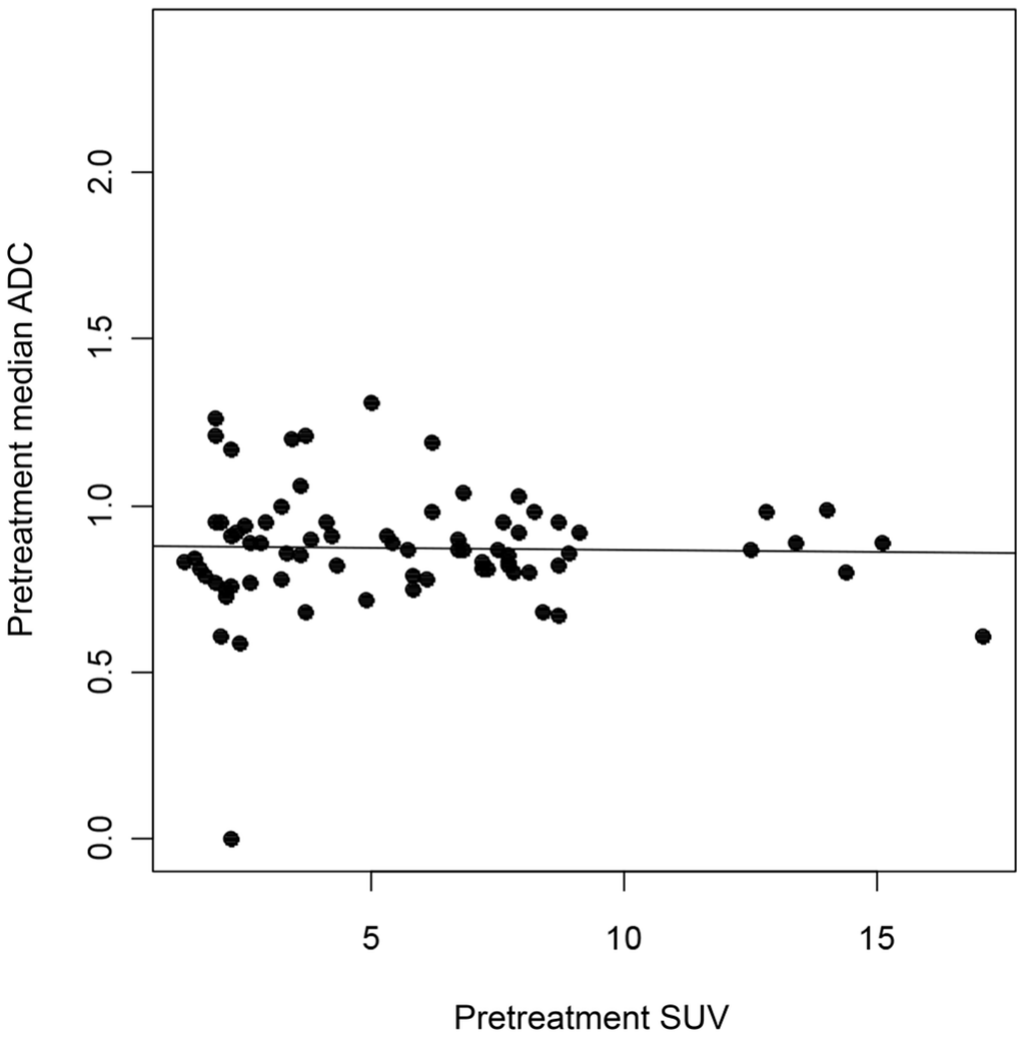
Dispersion of ADC and SUV in target lesions as observed in staging examinations. ADC = apparent diffusion coefficient, SUV = standard uptake value.

ADC and SUV were not significantly correlated. As such, lower ADC values do not necessarily imply higher SUV in the target lesions (Fig 1). There was no difference between primary tumors and lymph nodes.

### Treatment Response

Pre- and post-treatment comparisons of data pertaining to all target lesions revealed reductions in tumor size and SUV, as well as increases in ADC values, all of which were statistically significant.

The analysis of tumor size before and after treatment in patients who underwent both assessments (n = 19) revealed that the mean largest tumor dimension was 2.6 cm prior to treatment, and decreased to 1.8 cm after treatment. Mean ADC values increased from 0.89 x 10^−3^ mm^2^/s before treatment to 1.21 x 10^−3^ mm^2^/s after the intervention. Mean tumor SUV decreased from 5.68 to 3.54 after treatment. All differences between pre- and post-treatment measurements were statistically significant (*P* < .001).

**Fig 2:**
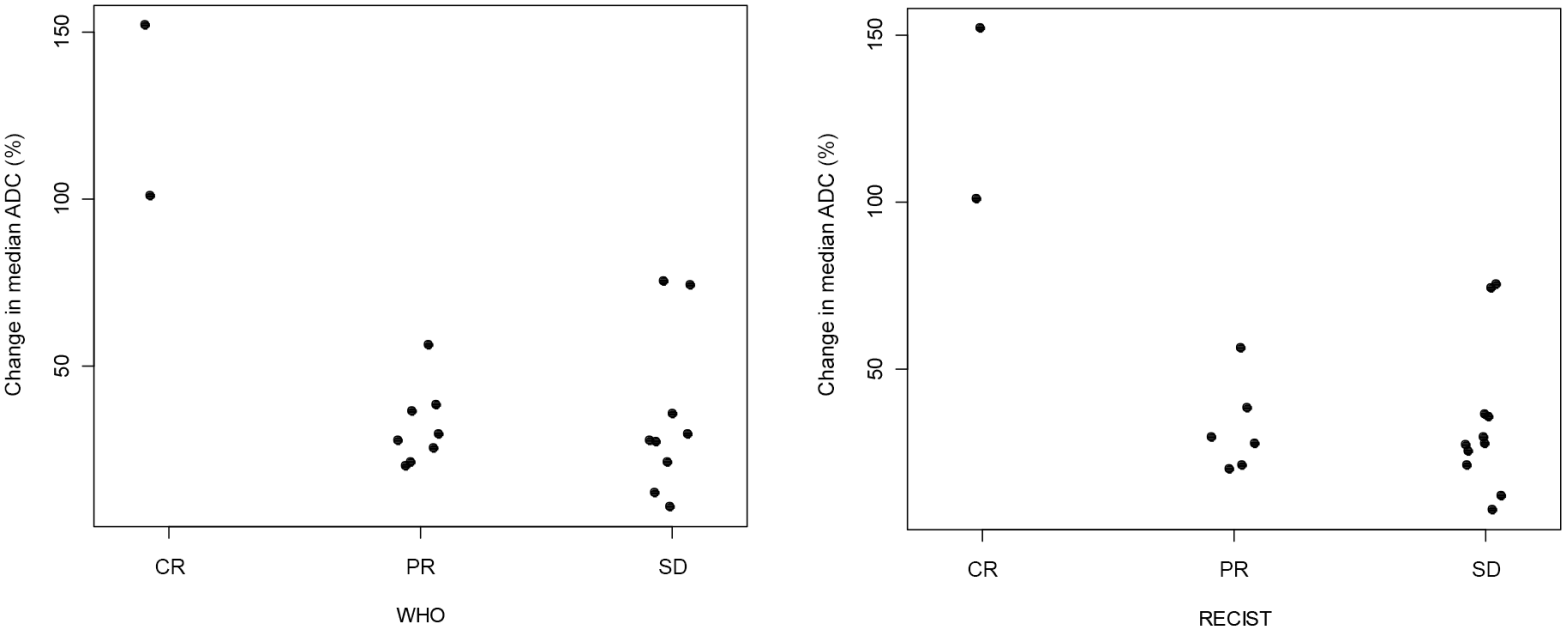
Correlation between changes in ADC values and treatment response categories according to WHO and RECIST guidelines. ADC = apparent diffusion coefficient, SD = stable disease, CR = complete response, RECIST = Response Evaluation Criteria In Solid Tumors, PR = partial response, WHO = World Health Organization.

Patients were also classified according to RECIST and WHO criteria based on the sum of the largest tumor diameters or that of the bidirectional product of the largest diameters, respectively. Two patients were classified as having PR according to WHO criteria but considered to have SD by RECIST. Therefore, according to RECIST, 11 patients were classified as having SD (57%), 6 as having PR (32%) and 2 as having CR (11%). According to WHO criteria, 9 patients had SD (47%), eight had PR (42%) and 2 had a CR (11%). The increase in ADC following treatment in patients classified as having a CR by both criteria was greater than 100%, and significantly higher than that observed in any of the other patient groups (p = 0.04). Changes in ADC did not differ between patients with SD and PR (Fig 2).

**Fig 3:**
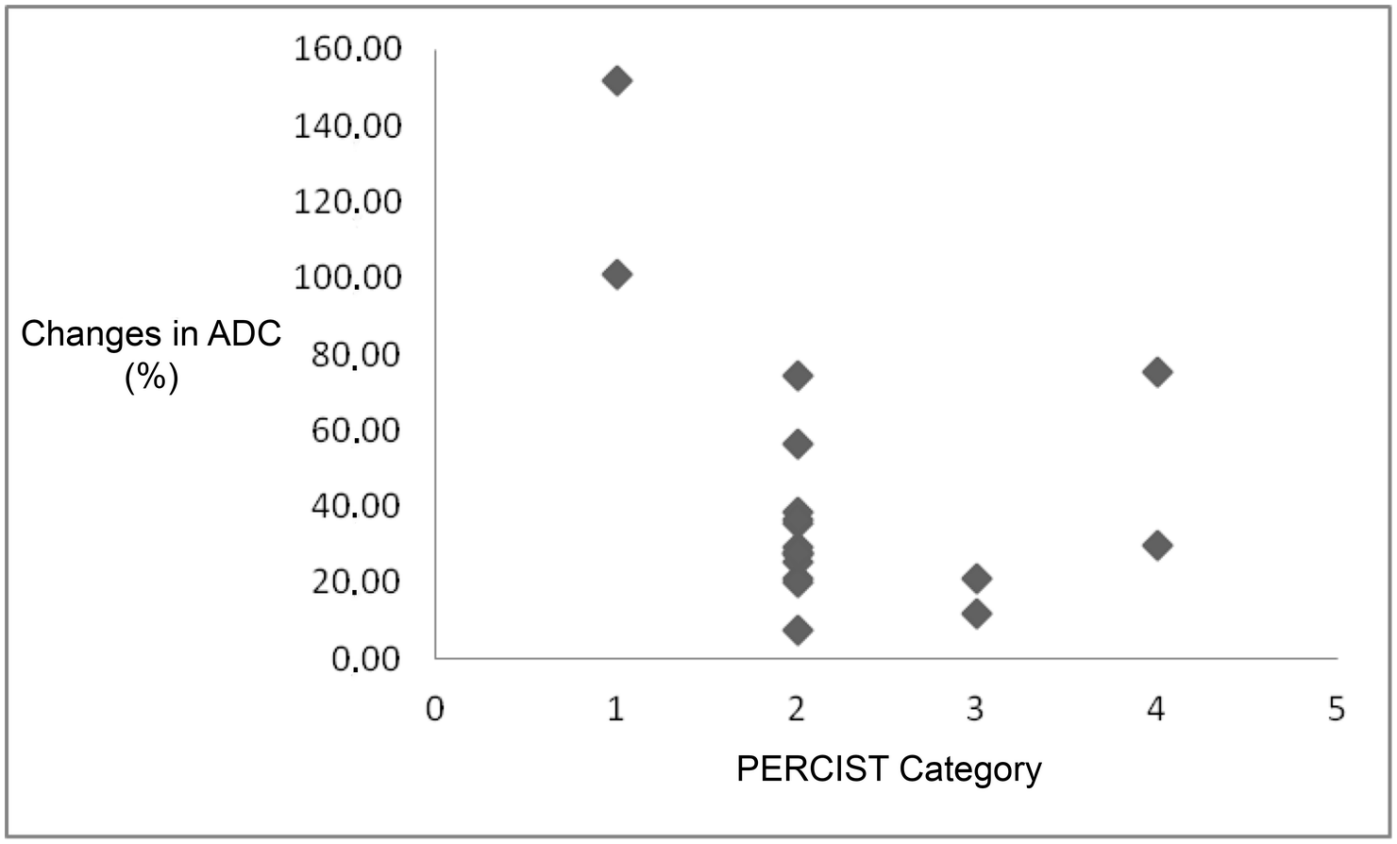
Correlation between changes in ADC values and treatment response categories according to SUV (PERCIST), where 1 indicates complete metabolic response, 2 corresponds to partial metabolic response, 3 to stable metabolic disease and 4 to progressive metabolic disease. ADC = apparent diffusion coefficient, SUV = standard uptake value, PERCIST = PET Response Criteria in Solid Tumors.

The classification of SUV according to PERCIST criteria indicated that two patients had a CMR (10.5%), 13 had a PMR (68.5%), two had SMD (10.5%) and another two had PMD (10.5%). Patients with a CMR showed greater increases in ADC values as compared to the remaining groups, whose changes in ADC following treatment did not differ from each other (Fig 3).

**Fig 4:**
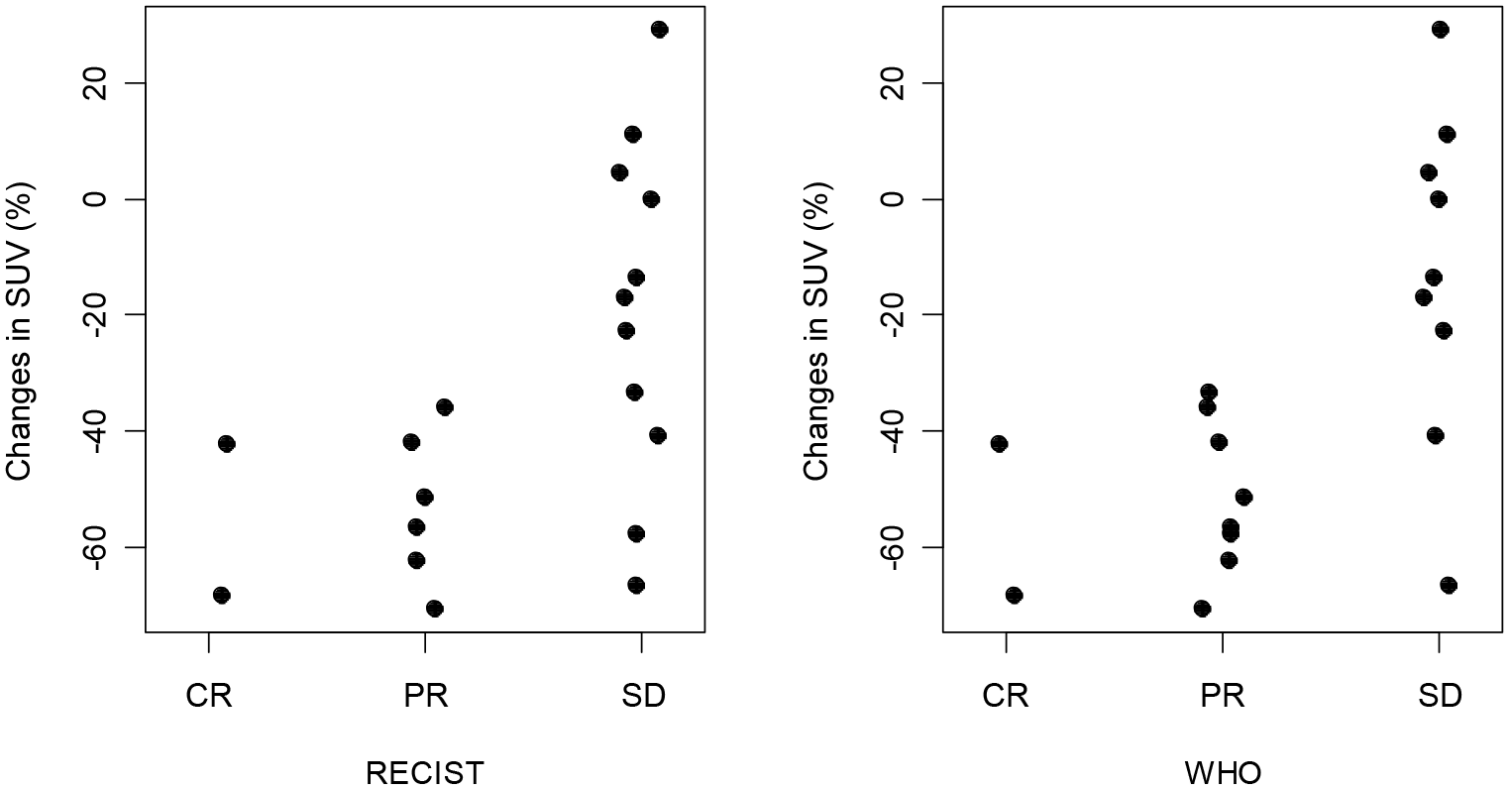
(A-B), Correlation between changes in SUV and treatment response according to WHO and RECIST guidelines. ADC = apparent diffusion coefficient, SD = stable disease, CR = complete response, RECIST = Response Evaluation Criteria In Solid Tumors, PR = partial response, SUV = standard uptake value, WHO = World Health Organization.

No between-group differences were observed in the percent reduction in SUV (relative to the mean lesion SUV per patient) or RECIST and WHO categories (Fig 4).

## Discussion

The present study evaluated the treatment response of patients with head and neck carcinoma using diffusion-weighted MRI, morphological criteria and [18F]-FDG PET/CT findings. Post-treatment evaluations showed significant increases in ADC, as well as reductions in SUV and tumor size. The comparison of morphological findings (WHO and RECIST) and ADC values revealed that patients with a CR showed greater increases in ADC values than the remaining participants. However, tumor SUV did not differ between treatment response groups. Patients with a CMR according to [18F]-FDG PET/CT (PERCIST) findings also showed significantly greater increases in ADC values following treatment.

The use of objective treatment response criteria is especially important to standardize findings and allow for comparisons between different study protocols and pharmacological treatments. Additionally, such criteria provide a non-invasive alternative for treatment monitoring in clinical practice, and may contribute to the identification and modification of ineffective treatments without the need of biopsies for histological analysis.

Park et al.[3] compared the two most widely used response criteria for the assessment of solid tumors (WHO and RECIST). Both sets of guidelines focus on changes in the size of the target lesions, although RECIST uses only the largest diameter of each tumor, while the WHO classifies patients according to the sum of the product of the two largest tumor diameters. Given the high agreement rates between the two criteria (91%), and the greater simplicity and reproducibility of RECIST guidelines, the authors suggested that these should be preferred over WHO criteria. In the present study, the two guidelines were also found to be highly concordant, except for two patients who were classified as having PR by WHO guidelines but considered to have SD by RECIST.

Therefore, there is a growing need for new and more effective techniques for the assessment of tumor response. Mean SUV decreased significantly from pre- to post-treatment, suggesting a decrease in the glucose metabolism of tumor cells following treatment. Ryan[11] found [18F]-FDG PET/CT to be more sensitive than CT alone in detecting the persistence of disease following radiotherapy. Yao et al.[12] showed that all post-radiotherapy patients with SUV below 3.0 were free from residual viable tumors. However, it is also important to note that SUV measurements may be influenced by variables such as the imaging system and its calibration, the dose of [18F]-FDG and time since irradiation, as well as serum glucose levels, so that SUV alone should not be used to evaluate tumor response. Additionally, radiation-induced alterations in inflammatory uptake may also lead to increases in SUV levels.[4] In the present study, patients were evaluated early in the induction chemotherapy cycle so as to assess tumor response in the first stage of treatment. This approach avoids false-positives caused by treatment-associated inflammatory changes.

Increased ADC values following radiotherapy and/or chemotherapy in patients with head and neck carcinoma have also been reported by several other authors, suggesting that diffusion measurements may be reliable early predictors of treatment outcome.

Vandecaveye et al.[13,14] evaluated treatment response after two years of follow-up using diffusion-weighted MRI and volumetric tumor measurements, classifying patients according to response to treatment and performing between-group comparisons of changes in ADC values and tumor volume as measured prior to treatment, as well as 2 and 4 weeks after the beginning of chemoradiotherapy. Complete responders showed a significantly greater increase in ADC values than those who showed post-treatment recurrence. Changes in tumor volume were not significantly associated with treatment response.

Kim et al.[15] analyzed the applicability of diffusion MRI as an early predictor of response to chemoradiotherapy, and found that patients with a favorable response had significantly lower ADC values in pre-treatment exams than those classified as partial or non-responders. Additionally, the variation in ADC values between pre-treatment and measures taken 1 week after the beginning of chemoradiotherapy was highly accurate in differentiating between complete and partial responders.

Choi et al.[16] were the first authors to publish a comparative study of diffusion MRI and [18F]-FDG PET/CT in head and neck carcinoma. The study found a high correlation between SUV and ADC as measured on both standard and high b-value diffusion. However, there is still a need for comparative studies of the effectiveness of diffusion measurements versus [18F]-FDG PET/CT in the assessment of treatment response. In the present study, the pattern of ADC changes differed significantly between patients with a CMR and those in other PERCIST categories, as assessed by [18F]-FDG PET/CT findings. However, the change in ADC values did not differ between the remaining groups. These findings may be attributable to the low number of patients with PMD and the fact that these measures are associated with distinct biological mechanisms: while diffusion is associated with cellularity, [18F]-FDG PET/CT evaluates glucose uptake.[17] Although several studies have confirmed the usefulness of both methods in assessing tumor response, few investigations have compared their relative efficacy or attempted to increase their precision.

The present findings agree with the recent literature on treatment response in patients with head and neck SCC, and underscore the importance of treatment assessment methods in oncology. In 2009, Padhani et al.[18] published a consensus report on the use of diffusion-weighted MRI in the assessment of tumor response, which resulted in an increased use of this method in research.

Our results showed that patients classified as complete responders according to morphological (WHO and RECIST) and functional (PERCIST) criteria showed greater post-treatment increases in ADC than the remaining patients groups. However, no between-group differences were found with regard to tumor SUV. These discrepancies suggest that morphological criteria may be unable to provide a precise assessment of treatment response, since they do not account for markers of disease activity such as cell proliferation and cellularity, which can be indirectly evaluated by both diffusion-weighted MRI and PET/CT.[16,19] Since these variables are not always related to changes in tumor size, morphological criteria may soon come to be replaced by functional assessments in both research and clinical practice. Discrepancies between patient classification according to [18F]-FDG PET/CT and diffusion-weighted MRI findings may be associated with differences between the biological parameters assessed by the two methods: while [18F]-FDG PET/CT is associated with glucose intake, diffusion-weighted MRI focuses more heavily on cell counts and tissue microarchitecture.[9] However, larger controlled studies with longer follow-up periods are still required to provide a definitive comparison between the two methods and identify which one may be more accurate in assessing treatment response.

Limitations of the present study include the small number of patients classified as having a CR or SD according to WHO and RECIST criteria. This may be due to the fact that patients were examined early in the treatment cycle, and that most were receiving neoadjuvant treatment. A definitive assessment of the precision of each guideline would require a study of the clinical evolution and survival rates of cancer patients over a longer period of time. This study did not include the response assessment at the end of treatment. Future studies should address the relation between early functional changes and actual response at the end of treatment.

In conclusion, diffusion-weighted MRI results showed that ADC levels increased significantly between pre- and post-treatment assessments in patients with head and neck SCC treated with chemotherapy and radiotherapy. Associations between early changes in ADC values and treatment response categories using morphologic criteria and [18F]-FDG PET/CT were only identified in complete responders.
